# Effects of different biomass materials as a salt-isolation layer on water and salt migration in coastal saline soil

**DOI:** 10.7717/peerj.11766

**Published:** 2021-07-07

**Authors:** Mao Yang, Runya Yang, Yanni Li, Yinghua Pan, Junna Sun, Zhenhua Zhang

**Affiliations:** 1 School of Resources and Environmental Engineering, Ludong University, Yantai, Shandong, China; 2 School of Life Sciences, Ludong University, Yantai, Shandong, China

**Keywords:** Infiltration rate, Phreatic evaporation, Water migration, Salt distribution, Salt-isolation

## Abstract

The aim of this study was to find a material suited for the prevention of evaporative water loss and salt accumulation in coastal saline soils. One-dimensional vertical water infiltration and phreatic evaporation experiments were conducted using a silty loam saline soil. A 3-cm-thick layer of corn straw, biochar, and peat was buried at the soil depth of 20 cm, and a 6-cm-thick layer of peat was also buried at the same soil depth for comparison. The presence of the biochar layer increased the upper soil water content, but its ability to inhibit salt accumulation was poor, leading to a high salt concentration in the surface soil. The 3-cm-thick straw and 6-cm-thick peat layers were most effective to inhibit salt accumulation, which reduced the upper soil salt concentration by 96% and 93%, respectively. However, the straw layer strongly inhibited phreatic evaporation and resulted in low water content in the upper soil layer. Compared with the straw layer, the peat layer increased the upper soil water content. Thus, burying a 6-cm-thick peat layer in the coastal saline soil is the optimal strategy to retain water in the upper soil layer and intercept salt in the deeper soil layer.

## Introduction

The groundwater quality in estuarine coastal zones, such as the Yellow River Delta in China, is mainly controlled by seawater intrusion and climate change such as frequent droughts and floods (Cui et al., 2021). Coastal groundwater typically occurs at shallow depths with high salinity. The salt in coastal groundwater tends to move with upward capillary water and accumulate in the surface soil, resulting in a wide distribution of saline soils over large areas. The saline soils are commonly characterized by poor physicochemical properties, low nutrient availability, and limited plant productivity (Luo et al., 2017; You et al., 2021; Zheng et al., 2020). The amelioration of coastal saline soils is essential to sustainable agriculture in coastal zones.

In the Yellow River Delta region, different soil amelioration methods, including engineering, biological, and agricultural measures, have been applied over the past decade (Mao et al., 2016; Shi et al., 2021). As a result, the soil salt content is decreased to 0.3% or less and the survival rate of plant seedlings reaches more than 95% in some coastal saline salt marshes (Li et al., 2012). Among the previously used methods, engineering measures require relatively high water conservancy investment, while biological measures exhibit slow effects based on the application of microbial agents (Luo et al., 2017; Yuan et al., 2019). Therefore, it is imperative to develop a fast and cost-effective method for the amelioration of coastal saline soils.

It was proposed by Jia et al. (2006) that the mulching material can be buried at an appropriate depth of 20–40 cm underground, which forms a capillary barrier layer to slow down salt accumulation in the root-growing zone of crops. Ityel et al. (2012) added a 5-cm-thick layer of gravel near the roots of pepper plants, which improved water use efficiency and, in turn, increased pepper yield by 40%. Additionally, Zhao et al. (2014) conducted a micro-plot experiment and found that burying a 5-cm-thick straw layer combined with plastic mulching effectively reduced salt content in the surface soil and increased sunflower yield; meanwhile, it improved soil water and heat conditions, while activating soil nutrients. Furthermore, Chen et al. (2016) found that the burying of a 3-cm-thick straw layer relieved salt stress and improved the quality and yield of tomatoes. Taken together, these studies demonstrate that the presence of a capillary barrier layer can effectively prevent salt accumulation and decrease soil salinity in the topsoil (Huang et al., 2011; Zhao et al., 2013; Zhao et al., 2016).

Previous studies mainly used carbon-rich crop straw as a capillary barrier layer (Zhao et al., 2018), mainly because of its high yields and abundant sources in agricultural production. However, if raw biomass materials, such as crop straw, are buried directly in the soil, it is easy to cause ‘burning’ (i.e., dehydration and wilting) of seedlings and nutrient (e.g., nitrogen) deficiency in the roots, negatively affecting crop growth (Martens, 2000; Li et al., 2020). Are there alternative salt-isolation materials that can be used to prevent salt accumulation from saline groundwater while increasing organic matter content in the upper soil layer? Finding such alternative materials is vital to maximize the benefits of the capillary barrier layer for coastal saline soils.

In recent years, peat and biochar have been increasingly used as soil amendments for improvement of soil fertility. Peat is rich in organic matter and humic acid, with a large specific surface area (Rezanezhad et al., 2016; Rezanezhad et al., 2017). More importantly, peat contains mature organic matter that will not generate fermentation heat to harm plant seedlings after application (Akhtar et al., 2019). Furthermore, biochar is a solid product produced by pyrolysis of biomass under anoxic or hypoxic conditions. It usually contains 40%–75% carbon and is characterized by high porosity, large specific surface area, and high ion exchange capacity (Cheng, Lehmann & Engelhard, 2008; Yuan et al., 2011a; Yuan et al., 2011b). However, most of the studies using peat and biochar as soil amendments have mixed the materials into the soil (Ghosh et al., 2006; Bezborodov et al., 2010). Further studies are still required to investigate the effects of burying peat and biochar at certain soil depths as a salt-isolation layer to conserve soil water and prevent salt accumulation in the surface soil.

Therefore, the present study was carried out to determine the effects of different salt-isolation materials (straw, peat, and biochar) on soil water and salt migration in a coastal saline soil using one-dimensional infiltration and evaporation experiments. The suitable material was selected and then used to optimize the thickness (3 and 6 cm) of salt-isolation layer. The aim of this study was to establish a method for improving the quality of saline soils in coastal zones in terms of water conservation and soil desalinization.

## Materials and Methods

### Experimental materials

The experimental soil was taken from the Yellow River Delta Coastal Wetland Ecological Experiment Station, Chinese Academy of Sciences in April 2019 (37°45′50″N, 118°59′24″E). At the sampling site, the plants were distributed in patches. The composition of the plant community was simple, mainly including salt-tolerant plants. The dominant plant species were *Suaeda salsa* (L.) *Pall*., *Phragmites communis* (Cav.) *Trin. ex Steud., Tamarix chinensis* Lour., and *Imperata cylindrica* (L.) Beauv. The soil sample was taken from a depth of 0–60 cm. The mass fraction of each particle size class in the soil was determined using a hydrometer. The soil contained 11.6% sand (0.02–2 mm), 48.7% silt (0.002–0.02 mm), and 39.7% clay (<0.002 mm), suggesting a silty loam texture. With regard to salt composition, the soil was classified as chloride-sulfate saline soil. Before the start of the experiment, the collected soil was air-dried and ground. After removing the debris, the soil was passed through a two mm sieve. The initial water content of the prepared soil was 1.04%, and the electrical conductivity of the soil solution was 1.9 mS/cm. The salt concentration was 4.8 g/kg, mainly containing Na^+^ and Cl^−^ ions (Zhang, Chen & Liu, 2019). The saturated water content measured by filling the soil into a cutting ring and saturating it with water was 40.18%. The field capacity measured by Wilkes’s method was 27.50%.

The straw used in this study was collected from corn field, air-dried, and cut into 3–5 cm lengths before use. Biochar was produced from corn straw by pyrolysis at 450 °C for 0.5 h. The biochar product had a pH of 8, with the organic matter content of 418 g/kg and the cation exchange capacity of 2.1 cmol/kg. Peat (residue of swamp plants) was purchased from Lusheng Biotechnology Co. Ltd. (Shouguang, Shandong, China), with a pH of 6.1, organic matter content of 682 g/kg, cation exchange capacity of 11.2 cmol/kg. The biochar and peat were passed through a two mm sieve before use.

### Experimental apparatus and design

**Figure 1:**
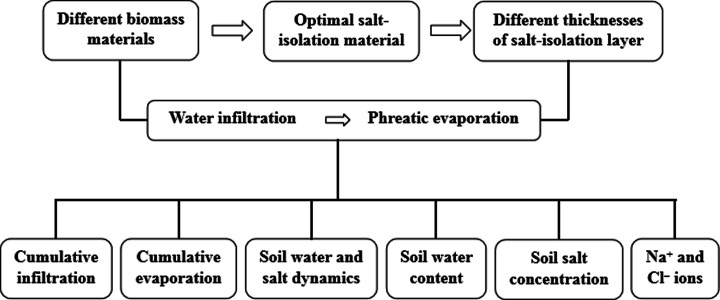
Flowchart of the experimental procedure.

**Figure 2:**
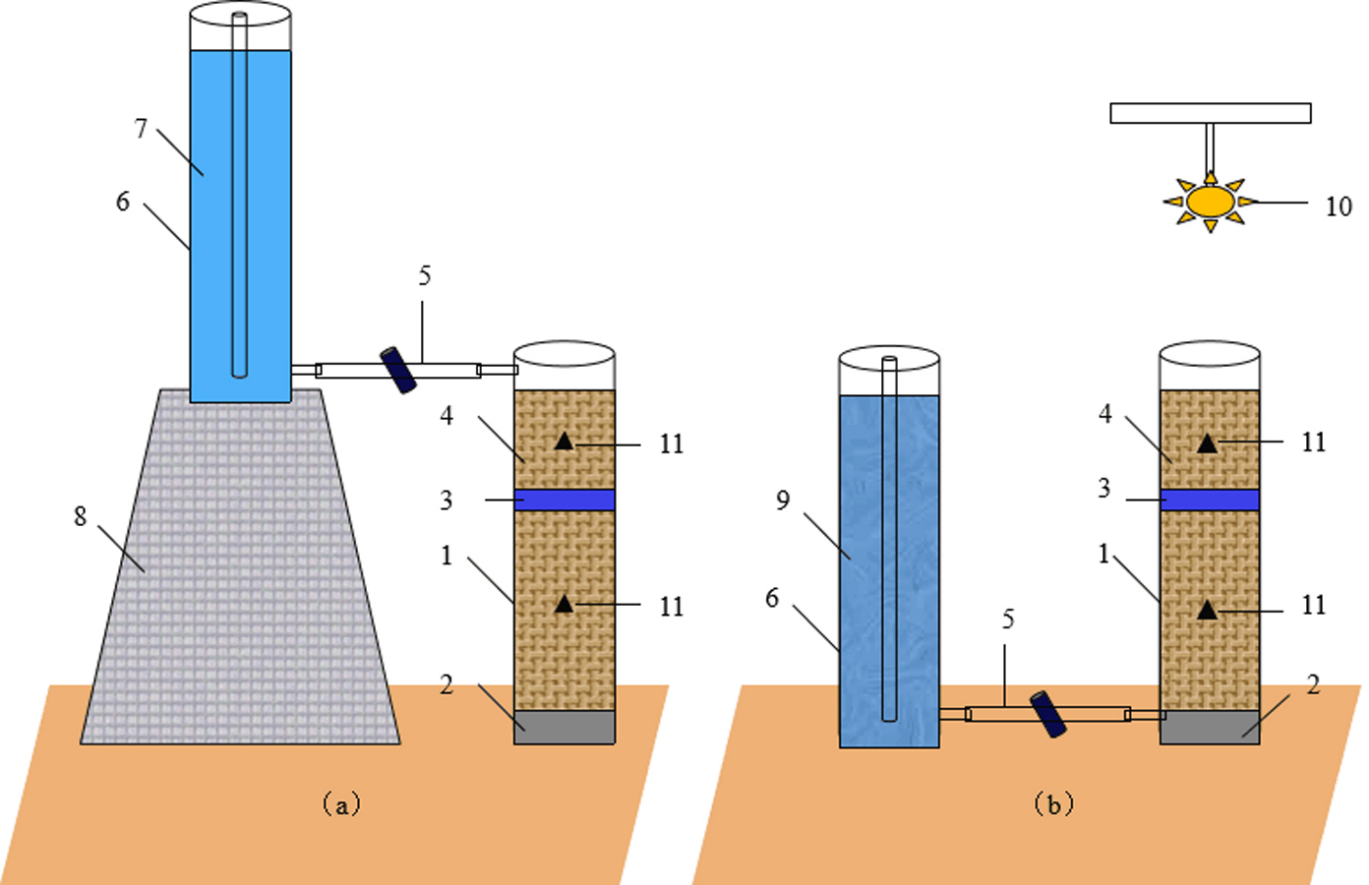
Schematic of the infiltration (A) and evaporation (B) experiments. (1) Soil column; Quartz sand; (3) Salt-isolation layer; (4) Homogeneous soil; (5) Water supply tube; (6) Marriotte’s bottle; (7) Deionized water; (8) Iron stand; (9) Simulated saline groundwater; (10) Infrared light; and (11) 5TE sensor.

A flowchart showing the experimental procedure is presented in Fig. 1. The apparatus used for the infiltration and evaporation experiments is shown in Fig. 2. The experimental apparatus consisted of a plexiglass column, a water supply bottle, and an iron stand. The plexiglass column had an inner diameter of 10 cm and a height of 90 cm. Before filling soil, Vaseline was evenly spread on the inner wall of the column to prevent the generation of preferential flow during infiltration. Then, a 10-cm-thick layer of quartz sand was added at the bottom of the column to prevent the outlet from clogging by soil particles. The experimental soil was filled into the column layer by layer (5 cm depth per layer), with a total depth of 50 cm. Water was supplied using a Marriotte’s bottle with an inner diameter of 9.8 cm and a height of 100 cm. The water head was controlled at 2 cm when supplying water.

The experiments tested five treatments (Table 1). In the control (Ctrl) treatment, the plexiglass column was filled with 50 cm of homogeneous soil only, while in the remaining four treatments, the plexiglass column was filled with 30 cm of homogeneous soil, followed by different salt-isolation materials (separated from the adjacent soil layers using filter paper), and then 20 cm of homogeneous soil. The thickness of salt-isolation layer was designed based on previous studies (Chen et al., 2016; Zhao et al., 2014; Zhao et al., 2016). To determine the effects of different salt-isolation materials, peat (P3), biochar (B3), and straw (S3) were added separately to form a 3-cm-thick layer each. To optimize the thickness of salt-isolation layer, peat was added to form 3-cm- and 6-cm-thick layers (P3 and P6, respectively). The bulk density of the soil in the column was set to 1.35 g/cm^3^. The bulk density of the salt-isolation materials added to the column was measured using a 100 cm^3^ cutting ring, i.e., 0.17 g/cm^3^ for peat, 0.45 g/cm^3^ for biochar, and 0.16 g/cm^3^ for straw. There were three replicates per treatment.

**Table 1:** Different treatments of soil column.

**Treatment**	**Description**
Control	50 cm homogeneous soil
S3	30 cm homogeneous soil + 3 cm straw + 20 cm homogeneous soil
B3	30 cm homogeneous soil + 3 cm biochar + 20 cm homogeneous soil
P3	30 cm homogeneous soil + 3 cm peat + 20 cm homogeneous soil
P6	30 cm homogeneous soil + 6 cm peat + 20 cm homogeneous soil

### Simulation experiments and data collection

After the soil column was filled, one-dimensional vertical infiltration experiment was carried out using deionized water. During the infiltration event, real-time monitoring of soil water content and electrical conductivity was carried out at 10 min intervals using an ECH_2_O-5TE sensor (Decagon Devices, Pullman, WA, USA), which was buried above (10 cm depth) and below (35 cm depth) the salt-isolation layer. The water level in Marriotte’s bottle and the depth of wetting front in sol column were observed at different time intervals (from short to long). Meanwhile, the cumulative infiltration was calculated. The infiltration experiment ended when the soil water content in the column reached the field capacity. The column was let stand for 2 days before the start of the phreatic evaporation experiment.

The evaporation experiment was performed using a 250 W infrared lamp as a light source, with the lower edge of the bulb at 30 cm above the soil surface. The duration of evaporation was 12 h per day (07:00–19:00). The initial groundwater depth in soil column was set to 50 cm. The Marriotte’s bottle was used to supply simulated saline water (containing 10 g/L NaCl and 10 g/L Na_2_SO_4_) and control the water level. Soil water content and electrical conductivity were measured as described for the infiltration experiment. Daily change in the water level of Marriotte’s bottle was recorded as the daily recharge (daily evaporation) of saline groundwater to the soil, and the cumulative recharge (cumulative evaporation) was obtained as the sum of daily recharge (daily evaporation). The evaporation event lasted 25 days.

At the end of the evaporation experiment, soil samples were taken from depths of 5, 15, 25, 35, and 45 cm. Soil water content was measured by weighing after oven-drying. In addition, the samples were air-dried and then soaked in deionized water (w/v, 1:5) to extract water-soluble salt. As the salt content of coastal saline soils in the Yellow River Delta is dominated by Na^+^ and Cl^−^ ions (Zhang, Chen & Liu, 2019), only the concentrations of water-soluble Na^+^ and Cl^−^ ions were measured in this study. Furthermore, salt-isolation materials before and after evaporation was air-dried and soaked in deionized water (w/v, 1:10), and the salt concentrations in the extract were analyzed by measuring electrical conductivity. The measurement indicators and methods are summarized in Table 2.

**Table 2:** The indicators and methods of experimental measurements.

**Indicator**	**Method**
Soil water and salt dynamics	ECH_2_O-5TE sensor (Decagon Devices, Pullman, WA, USA)
Soil water content	Oven drying at 105 °C and weighing (Lu, 2000)
Soil salt concentration	Residue drying and weighing (Lu, 2000)
Water-soluble Na^+^	Flame photometry (Lu, 2000)
Water-soluble Cl^−^	Silver nitrate titration (Lu, 2000)
Soil electrical conductivity of salt-isolation material	Electrical conductivity measurement (Lu, 2000)

### Data analysis

During the 25-day evaporation experiment, the average increase of water content (Δ_Water_) and salt concentration (Δ_Salt_) in the 0–20 cm soil layer for every 1,000 mL of saline groundwater evaporation was calculated using empirical equations modified from Zhu et al. (2019):

Δ_Water_ (%) = (Final water content − initial water content) / Cumulative evaporation * 1000.

Δ_Salt_ (g/kg) = (Final salt concentration − initial salt concentration) / Cumulative evaporation * 1000.

Statistical analysis was performed using SPSS Statistics 18.0 (SPSS Inc. Chicago, IL, USA). One-way analysis of variance (ANOVA) followed by Duncan’s test was used to determine significant differences among the treatments (*p* < 0.05).

## Results

### Effects of different salt-isolation treatments on water infiltration characteristics

#### Wetting front migration

**Figure 3:**
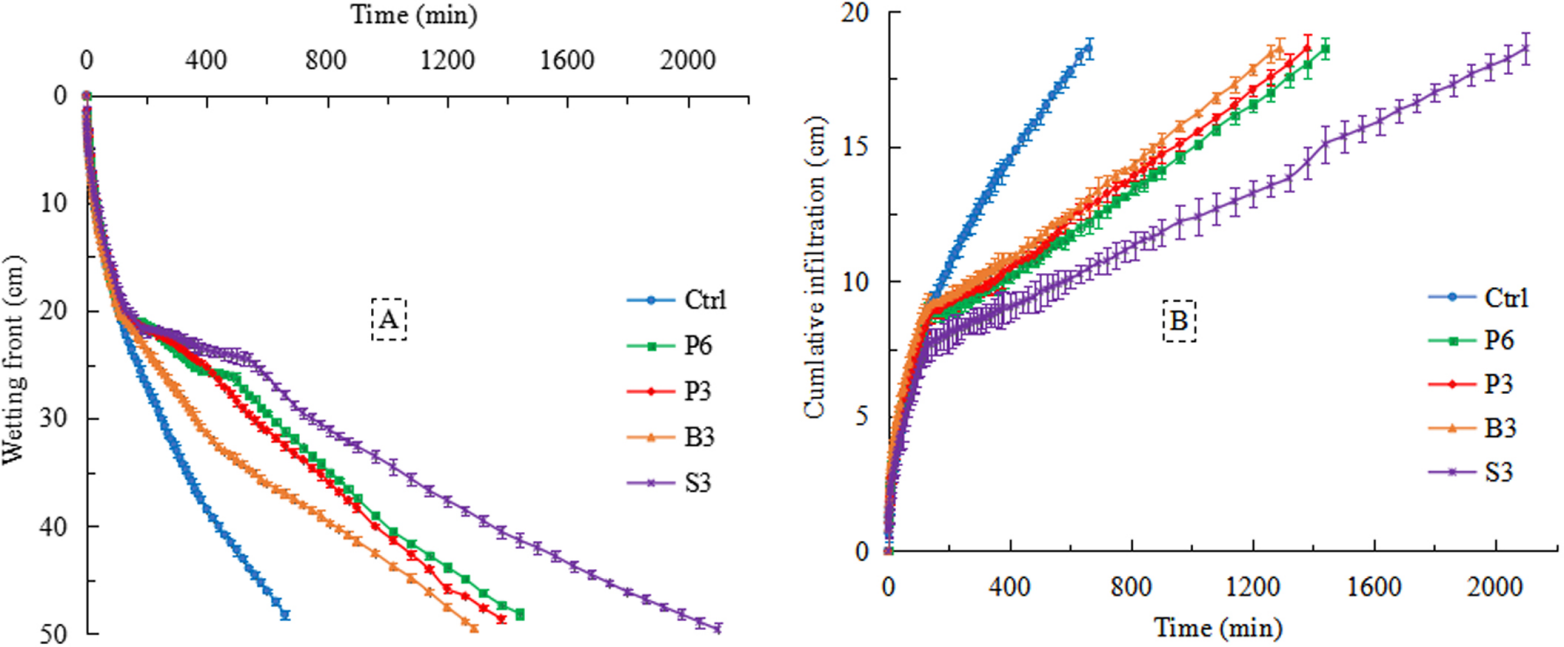
Changes in the migration distance of wetting front (A) and the cumulative infiltration (B) with infiltration time under different treatments. Ctrl, control without salt-isolation material; S3, addition of a 3-cm-thick straw layer; B3, addition of a 3-cm-thick biochar layer; P3, addition of a 3-cm-thick peat layer; P6, addition of a 6-cm-thick peat layer.

Before the wetting front migrated to the salt-isolation layer (20 cm), the infiltration duration of different treatments was close, and the advance velocity of the wetting front was generally identical (Fig. 3A). With increasing time of infiltration, the salt-isolation layer was penetrated by preferential flow, and the corresponding time of salt-isolation treatments was ranked in the order: P6 (370 min) >S3 (220 min) >P3 (175 min) >B3 (50 min). This resulted in a nonuniform pattern of the wetting front, which gradually disappeared with increasing depth of infiltration. Among the different treatments, the infiltration duration of S3 treatment was the longest, which increased by 218% (*p* < 0.05) compared with that of Ctrl (660 min). The infiltration duration of P6 and P3 treatments was the second-longest (118% and 109%), while B3 treatment had the shortest infiltration duration (95%; *p* < 0.05).

#### Cumulative infiltration

From the start of infiltration to ∼110 min (before the wetting front reached the salt-isolation layer), the cumulative infiltration curves of different treatments were consistently steep (Fig. 3B). With increasing time of infiltration (>110 min), all salt-isolation treatments reduced water infiltration rate compared with the control. For example, at the time point of 600 min, the cumulative infiltration of Ctrl was the largest (18 cm), which was significantly higher than that of P6, P3, and B3 treatments (∼12 cm). The largest decrease of cumulative infiltration was observed for S3 treatment (43%).

### Water and salt distribution in the upper soil layer

**Figure 4:**
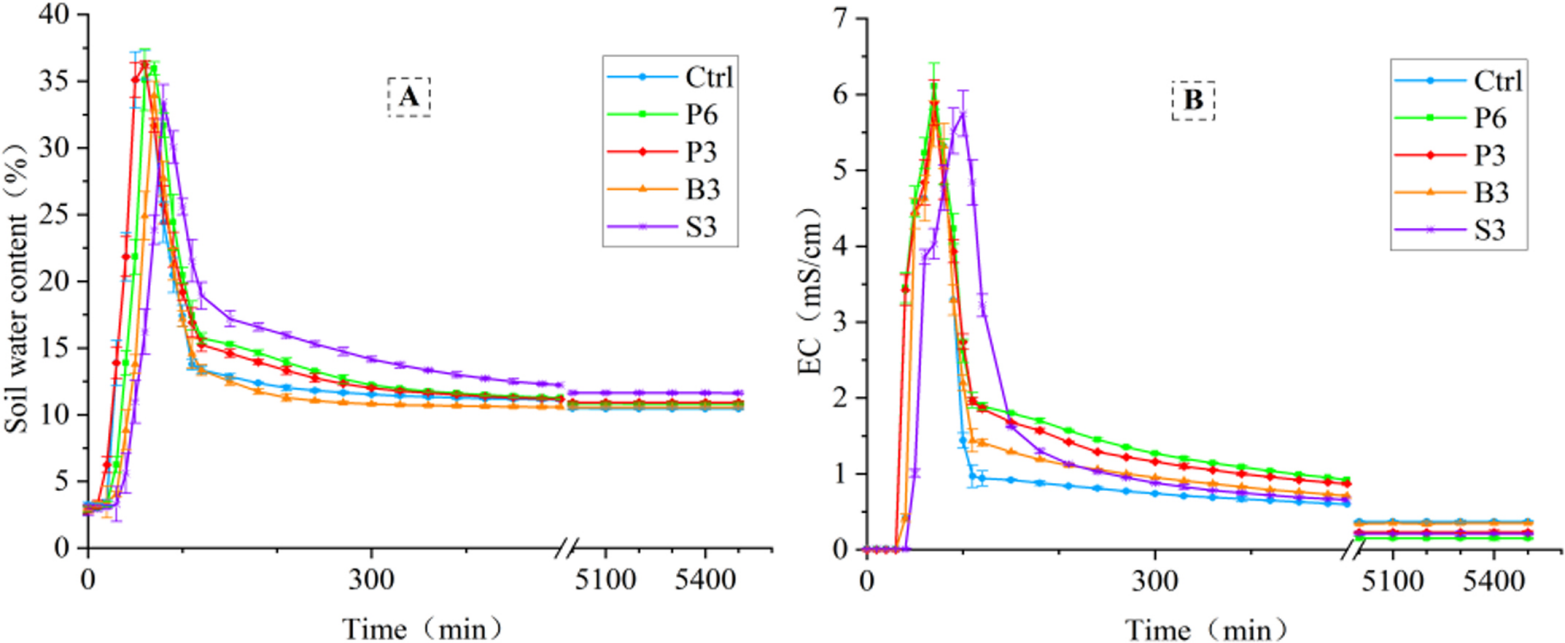
Changes in the 0–20 cm soil water content (A) and electrical conductivity (B) with infiltration time under different treatments. Ctrl, control without salt-isolation material; S3, addition of a 3-cm-thick straw layer; B3, addition of a 3-cm-thick biochar layer; P3, addition of a 3-cm-thick peat layer; P6, addition of a 6-cm-thick peat layer.

With the advance of the wetting front, the upper soil water content reached its peak value (Fig. 4A). As water gradually moved downward, the upper soil water content tended to level off. In the initial stage of leaching and infiltration (e.g., 110 min), the 0–20 cm soil water content of S3 treatment had the largest increase (56%) compared with that of Ctrl (14%), while moderate increases occurred in P6 and P3 treatments (27% and 23%, respectively). Further analysis revealed that the time period from the peak value to evident decrease of 0–20 cm soil water content increased by 213% in S3 treatment compared with Ctrl (150 min). In P6 and P3 treatments, this time period increased by 73% and 67%, respectively. These three salt-isolation treatments prolonged the retention time of soil water above the salt-isolation layer. However, no significant difference was found in the time period between B3 and Ctrl. In the later stage of leaching and infiltration (e.g., 5100 min), the 0–20 cm soil water content of S3 treatment increased by 11% compared with Ctrl (10%), while no significant differences were found between the remaining treatments.

As the wetting front advanced, salt gradually accumulated in the upper soil layer, with the electrical conductivity reaching a peak value (Fig. 4B). Subsequently, the upper soil electrical conductivity gradually decreased. In the later stage of leaching and infiltration, the upper soil electrical conductivity of all salt-isolation treatments was lower than that of the control. For example, at the time point of 5100 min, the 0–20 cm soil electrical conductivity of P6 treatment was 59% lower than that of Ctrl (0.4 mS/cm), while significant decreases of electrical conductivity also occurred in P3 and S3 treatments, 38% and 43%, respectively (*p* < 0.05).

### Effects of different salt-isolation treatments on phreatic evaporation characteristics

#### Daily evaporation

**Figure 5:**
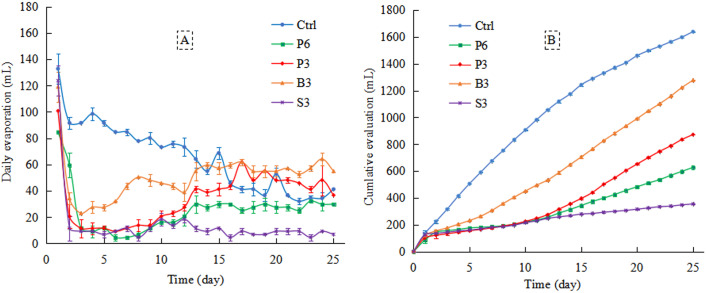
Changes in daily evaporation (A) and cumulative evaporation (B) with time under different treatments. Ctrl, control without salt-isolation material; S3, addition of a 3-cm-thick straw layer; B3, addition of a 3-cm-thick biochar layer; P3, addition of a 3-cm-thick peat layer; P6, addition of a 6-cm-thick peat layer.

The highest daily evaporation was recorded on the 1st day of the evaporation process, >100 mL in all cases (Fig. 5A). From the 2nd to the 15th day, the daily evaporation of different treatments was ranked as follows: Ctrl > B3 > P3 > P6 > S3. Between the 16th and 25th days, the daily evaporation of B3 and P3 treatments gradually exceeded that of Ctrl. Averaged over the 25-day evaporation period, the daily evaporation of Ctrl was the highest (66 mL), followed by that of B3 (51 mL), while significantly lower daily evaporation was recorded for P3 (35 mL), P6 (25 mL), and S3 (14 mL; *p* < 0.05).

#### Cumulative evaporation

The cumulative evaporation of P6, P3, and S3 treatments did not significantly differ within the first 12 days of evaporation (Fig. 5B). After the salt-isolation layer was penetrated by phreatic water, the differences between P6, P3, and S3 treatments gradually emerged between the 13th and 25th days of evaporation, and the average cumulative evaporation was ranked in the order: P3 > P6 > S3. After 25 days of continuous evaporation, the cumulative evaporation of S3 treatment decreased the most (78%) compared with Ctrl (1639 mL). Moderate decrease of cumulative evaporation was observed in P6 and P3 treatments (62% and 47%, respectively), while the smallest decrease occurred in B3 treatment (22%).

#### Dynamic distribution of water and salt in the upper soil layer

**Figure 6:**
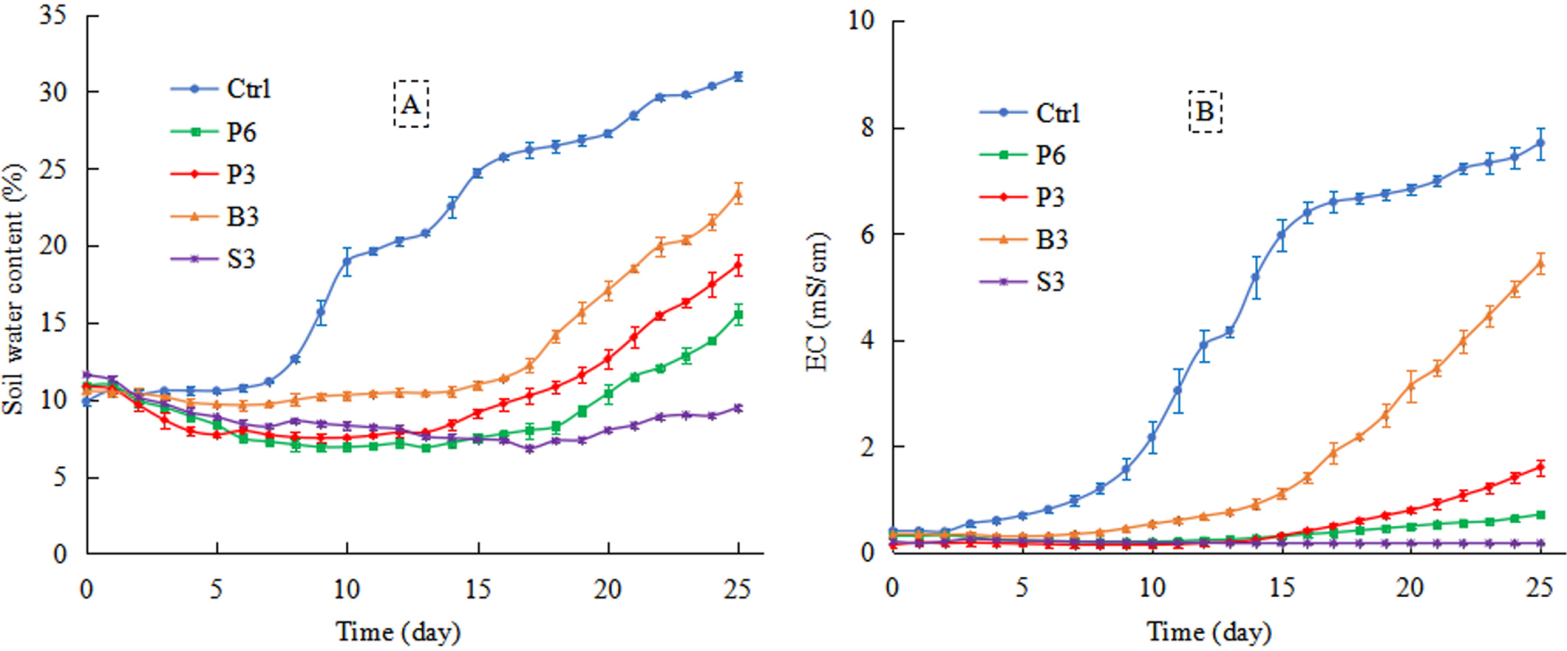
Changes in the 0–.20 cm soil water content (A) and electrical conductivity (B) with evaporation time under different treatments. Ctrl, control without salt-isolation material; S3, addition of a 3-cm-thick straw layer; B3, addition of a 3-cm-thick biochar layer; P3, addition of a 3-cm-thick peat layer; P6, addition of a 6-cm-thick peat layer.

Within the first 15 days of evaporation, there were no considerable differences among P6, P3, and S3 treatments in terms of the upper soil water content (Fig. 6A). After phreatic water penetrated the salt-isolation layer, the upper soil water content of P6 and P3 treatments increased rapidly from the 16th to the 25th day of evaporation, which was significantly higher than that of S3 treatment. Compared with Ctrl, salt-isolation treatments significantly decreased the upper soil water content. Across the 25-day evaporation period, the average upper soil water content of different treatments was ranked as Ctrl (20%) >B3 (13%) >P3 (10%) >P6 (9%) >S3 (8%).

Within the first 13 days of evaporation, the upper soil electrical conductivity of P6, P3, B3, and S3 treatments occurred at low levels, <1 mS/cm (Fig. 6B). After 13–25 days of evaporation, the salt-isolation layer was penetrated by phreatic water in P3 and B3 treatments, where salt gradually accumulated in the upper soil layer and the electrical conductivity increased continuously. During the 25-day evaporation period, the average upper soil electrical conductivity of Ctrl reached 3.9 mS/cm, while the average values of salt-isolation treatments were significantly lower, 0.4 mS/cm for P6, 0.5 mS/cm for P3, 1.6 mS/cm for B3, and 0.2 mS/cm for S3.

### Effects of different salt-isolation treatments on soil water and salt distribution after evaporation

**Figure 7:**
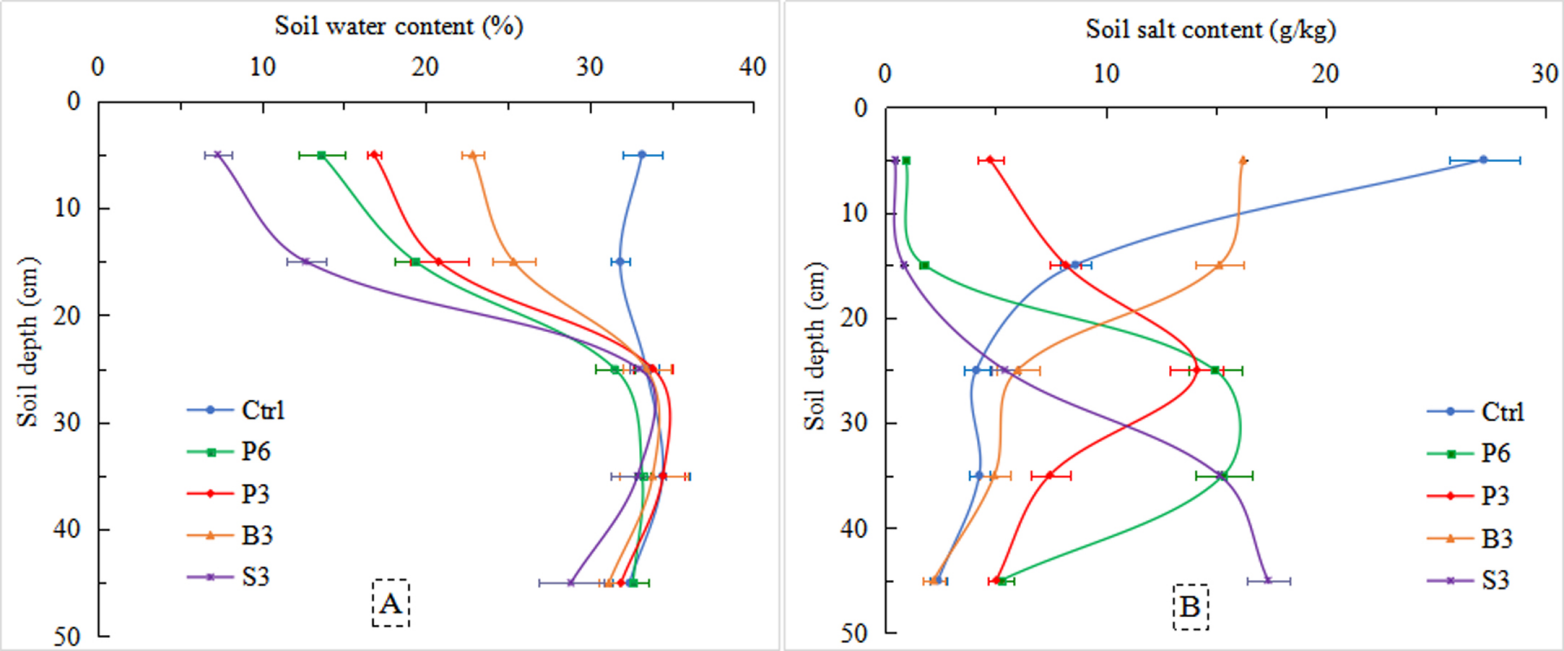
Soil water content (A) and salt concentration (B) in the soil profile after phreatic evaporation under different treatments. Ctrl, control without salt-isolation material; S3, addition of a 3-cm-thick straw layer; B3, addition of a 3-cm-thick biochar layer; P3, addition of a 3-cm-thick peat layer; P6, addition of a 6-cm-thick peat layer.

At the end of the evaporation experiment, there were no significant differences in the 20–50 cm soil water content (32–33%) below the salt-isolation layer among various treatments (Fig. 7A). However, the average water content in the 0–20 cm soil layer of B3 (23%), P3 (19%), and P6 (17%) was significantly higher than that of S3 treatment (10%; *p* < 0.05). Meanwhile, the average salt concentration in the 0–20 cm soil layer of Ctrl was the highest (17.9 g/kg; Fig. 7B). In the presence of a salt-isolation layer, the 0–20 cm soil salt concentration significantly decreased and varied with different materials or thickness of salt-isolation layer, B3 (15.7 g/kg) >P3 (6.5 g/kg) >P6 (1.3 g/kg) >S3 (0.6 g/kg). The largest decrease of salt concentration was recorded for S3 treatment (96%), followed by P6 treatment (93%). In the 20–50 cm soil layer, the average salt concentration of Ctrl was 3.6 g/kg. Except for B3, all the remaining salt-isolation treatments significantly increased the average soil salt concentration compared with Ctrl, 2.3-fold for P6, 1.5-fold for P3, and 2.5-fold for S3.

For every 1,000 mL of saline groundwater evaporation, the Δ_Water_ in the 0–20 cm soil layer of Ctrl was the highest, reaching 13.5% (Table 3). The Δ_Water_ values of P6, P3, and B3 treatments were close, at ∼9%, while the Δ_Water_ of S3 treatment was 0. Meanwhile, the highest Δ_Salt_ values were obtained from Ctrl and B3 (10.5 and 11.8 g/kg respectively), whereas P3 and P6 had significantly lower Δ_Salt_ (7.4 and 1.1 g/kg, respectively). The results showed that in the 0–20 cm soil layer, despite similar increase of water content among P6, P3, and B3 treatments, the corresponding increase of salt was minimal in P6 treatment.

**Table 3:** Increases of 0–20 cm soil water content and salt concentration for every 1,000 mL of saline groundwater evaporation.

	Ctrl	S3	B3	P3	P6
Water content (%)	13.5a	0	9.6b	8.5b	9b
Salt concentration (g/kg)	10.5a	0	11.8a	7.4b	1.1c

**Notes.**

Ctrl control without salt-isolation material S3 addition of a 3-cm-thick straw layer B3 addition of a 3-cm-thick biochar layer P3 addition of a 3-cm-thick peat layer P6 addition of a 6-cm-thick peat layer

At the end of the evaporation experiment, both the peat and biochar layers exhibited a salt interception effect (Table 4). Compared with their raw materials, the electrical conductivity of the peat and biochar layers after evaporation increased by 22.2- and 3.1-fold, respectively, indicating that the peat layer performed better in salt retention. The straw layer generally had no effect on salt retention, and there was no significant difference in its electrical conductivity compared with that of the raw material.

**Table 4:** Changes in electrical conductivity of salt-isolation materials before and after evaporation.

Salt-isolation material	Electrical conductivity (µS/cm)	
	Before evaporation	After evaporation
Straw	361b	372b
Biochar	61d	249c
Peat	24e	556a

**Notes.**

Values followed by different lowercase letters are statistically significant (*p* < 0.05)

### Effects of different salt-isolation treatments on soil Na^+^ and Cl^−^ concentrations after evaporation

**Figure 8:**
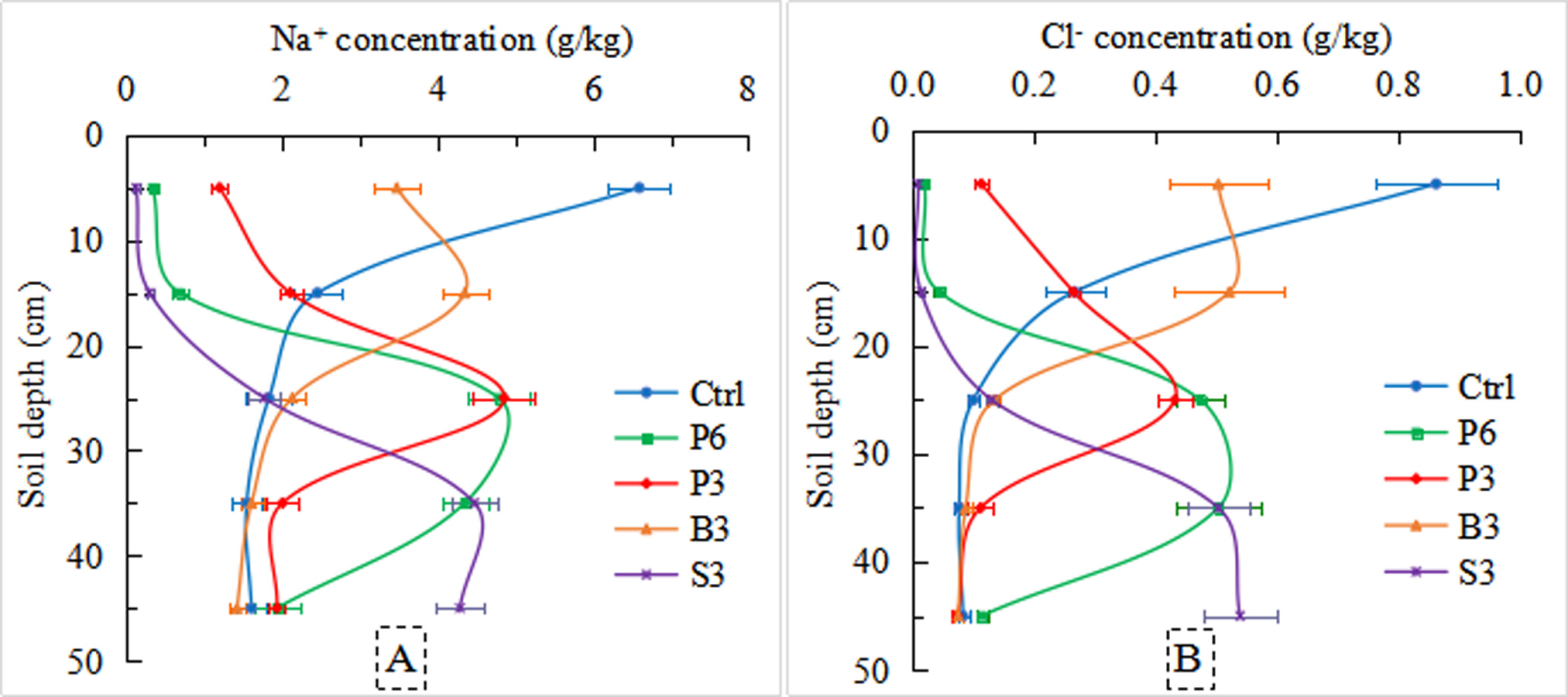
Distribution of Na^+^ (A) and Cl^−^ (B) ions in the soil profile under different treatments. Ctrl, control without salt-isolation material; S3, addition of a 3-cm-thick straw layer; B3, addition of a 3-cm-thick biochar layer; P3, addition of a 3-cm-thick peat layer; P6, addition of a 6-cm-thick peat layer.

In the 0–20 cm soil layer, the average Na^+^ concentrations of various treatments were ranked as Ctrl (4.5 g/kg) >B3 (3.9 g/kg) >P3 (1.7 g/kg) >P6 (0.5 g/kg) >S3 (0.2 g/kg; Fig. 8A). Among the different salt-isolation treatments, P6 and S3 resulted in the largest decrease in Na^+^ concentrations compared with Ctrl. In the 20–50 cm soil layer, the average Na^+^ concentrations of P6, P3, B3, and S3 treatments increased by 121%, 76%, 3.6%, and 110%, respectively, compared with Ctrl (1.7 g/kg). Similar changes were observed in the average Cl^−^ concentrations (Fig. 8B). Overall, in the S3, P6, and P3 treatments, the 0–20 cm soil Na^+^ and Cl ^−^ concentrations were relatively low, as the Na^+^ and Cl^−^ ions carried by saline groundwater were effectively intercepted below the salt-isolation layer.

## Discussion

### Effects of salt-isolation layer on water infiltration process in the saline soil

In this study, straw, peat, and biochar was buried separately as a salt-isolation layer in vertical soil column, which reduced water infiltration rate in the coastal saline soil (Fig. 3). Our result is consistent with the previous finding of Zhao et al. (2013) who treated a silt loam soil with straw as the salt-isolation material in Inner Mongolia. This is mainly because the presence of a salt-isolation layer altered soil permeability and broke the continuity of soil hydraulic conductivity, leading to an abrupt change in soil water potential at the interface between the soil and salt-isolation layer (Franzluebbers, 2002; Zhang et al., 2006). The ‘differential pore interface’ formed at the interface between fine-textured soil and coarse-textured salt-isolation material resulted in a difference in soil hydraulic conductivity (Li, Chang & Salifu, 2014; Li et al., 2018). As a result, the water flux entering the salt-isolation layer per unit time was reduced, which in turn lowered the infiltration rate of soil water. In the initial stage of the infiltration process, preferential flow tended to occur below the salt-isolation layer. As the infiltration process went on, the water content of salt-isolation layer reached a certain level and its hydraulic conductivity was close to that of fine soil; thus, the water infiltration rate became stable, and the preferential flow gradually disappeared.

As the wetting front advanced, the salt in the upper soil layer was leached to the depth of soil infiltrated by deionized water. The salt dynamics can be divided into two stages: formation of a salt peak in the surface soil and downward shift of the salt peak toward the deeper soil (Sun et al., 2019). During the leaching and infiltration process, salt-isolation treatment with straw and peat was found to facilitate desalinization of the upper soil layer. This is mainly due to the fact that in a homogeneous soil, the infiltrating water could enter the deeper layer before an equilibrium of water and salt diffusion was reached in the soil, so the salt-leaching effect of water per unit volume was poor (Akhtar, Andersen & Liu, 2015). In the presence of a peat or straw layer, the ponding time of infiltration water in the soil above the salt-isolation layer was prolonged (Fig. 4A), which might in turn promote ion exchange, adsorption, and desorption (Pang et al., 2010; Mavi et al., 2012; Wang et al., 2017). Consequently, the salt in the surface soil was fully dissolved and leached out by water. This might explain the relatively low salt concentrations in the soil treated with straw and peat during the later stage of infiltration (Fig. 4B).

### Effects of salt-isolation layer on phreatic evaporation process in the saline soil

In the early stage of phreatic evaporation process, the cumulative evaporation was markedly reduced by various salt-isolation treatments compared with the control. However, after 15 days of evaporation, the daily evaporation of both biochar (B3) and peat (P3) treatments was higher than that of homogeneous soil (Ctrl), along with considerably increased soil water content and electrical conductivity (Fig. 5A). This result indicates that in the later stage of evaporation, groundwater gradually penetrated the biochar and peat layers of 3 cm thickness, partly restoring the soil water migration channel (Simhayov, Weber & Price, 2018) and thus resulting in an increase in the upper soil water content (Fig. 6A). Meanwhile, the total salt, Na^+^, and Cl^−^ concentrations in the upper soil layer were increased (Figs. 6B and 8). However, the cumulative evaporation of salt-isolation treatments was 22.08%–78.35% lower than that of the control (Fig. 8). Indeed, after the rapid loss of water from most of the pores in the salt-isolation layer, an impervious layer could be formed (Li et al., 2016; Wang et al., 2019). When the capillary water from deeper soil reached the impervious layer, it would be difficult to pass and further move up (Zhao et al., 2014; Zhao et al., 2016; Zhang et al., 2020), thus reducing the evaporation intensity.

Among the different salt-isolation treatments, the straw layer performed the best in preventing salt accumulation in the coastal saline soil (Fig. 7B). Previously, Zhao et al. (2013) also found that a 5-cm-thick straw layer buried in the soil was effective to prevent evaporation and control resalinization in the Hetao Irrigation Distract in Inner Mongolia, China. However, it should be noted that there was no increase of water in the 0–20 cm soil layer for every 1000 mL of saline groundwater evaporation in the straw treatment (Δ_Water_ = 0) (Table 3). This result is unfavorable for water uptake by plant roots. A plausible reason is that the surface of the straw is smooth and dense, so that an impervious layer was formed after water loss from pores, and the strong blocking effect resulted in extremely low capillarity during evaporation. Therefore, the water from deeper soil could only diffuse in the form of water vapor (Zhao et al., 2013; Rao & Rekapalli, 2020), leading to low water content and salt concentration in the surface soil.

Although the biochar treatment increased upper soil water content, its effect to inhibit salt accumulation was poor. At the end of the evaporation process, the soil salt concentration of biochar treatment was the highest among the three different materials. This might be related to the porous structure of biochar, which created fewer macros pores and more effective pores in the soil (Xiao et al., 2016). As the evaporation progressed, the impervious layer formed after water loss from pores in the biochar could partially cut off soil capillarity and establish a semi-continuous channel for water migration. Therefore, biochar is not an ideal material for the inhibition of salt accumulation in saline soil.

In the peat treatments, with an increase in the thickness of salt-isolation layer, the salt concentration in the surface soil layer decreased significantly. This result indicates that the thicker the peat layer, the greater the effect for the prevention and control of evaporation salinization. Clearly, a thicker salt-isolation layer means a greater difficulty for the phreatic water to break through the salt-isolation material to rise. Since the soil salt moved upward with the soil water during evaporation, the salt concentration in the surface soil layer was relatively low, which is in agreement with the results of previous studies (Chen, Mao & Shukla, 2020; Zhang et al., 2020; Zhang et al., 2020). In addition, it was found that the upper soil water content of the 6-cm-thick peat treatment was almost the same as that of the biochar treatment. However, the upper soil salt concentration (Table 3), as well as the corresponding Na^+^ and Cl^−^ concentrations (Fig. 8), was substantially reduced in the 6-cm-thick peat treatment compared with the biochar treatment. Due to the microporous structure of the peat, this material could absorb part of the salt (Wang et al., 2014; Rezanezhad et al., 2016; Rezanezhad et al., 2017). This mechanism was supported by the relatively high salt concentration measured in the peat layer (Table 4). Simhayov, Weber & Price (2018) also found that peat played a role in water conservation, water storage, and salt absorption. Therefore, adding peat as a salt-isolation layer (6 cm thick) is the most effective way to retain water and facilitate desalinization in the topsoil, which could create a favorable soil environment for the growth of crop roots.

This study was based on soil column simulation experiments in the laboratory, so the results still need to be further verified by field experiments. In the future research, other factors, such as atmospheric precipitation and irrigation, should be taken into account, and the next step of our research is to couple more factors with crop growth to better understand their effects salt-isolation material on soil water and salt in coastal saline soils.

## Conclusions

This study demonstrated the effects of straw, biochar, and peat as salt-isolation materials on water and salt distribution in a coastal saline soil through soil column simulation experiments. It was found that the presence of a salt-isolation layer changed the temporal and spatial distribution patterns of water and salt in the soil profile. In particular, the salt carried by saline groundwater was intercepted in the deeper soil below the salt-isolation layer. Among the different salt-isolation materials tested, the straw layer performed the best in controlling salt accumulation; however, it strongly inhibited phreatic evaporation and resulted in low water content in the upper soil layer, which was not conducive to root growth. Compared with the control, the biochar layer increased the upper soil water content, yet its ability to inhibit salt accumulation was poor, leading to relatively high salt concentrations in the surface soil. The 6-cm-thick peat layer tended to prevent both phreatic evaporation and salt accumulation, which could intercept salt in the deeper soil. Compared with the straw layer, the peat layer also increased the upper soil water content. Thus, burying a 6-cm-thick layer of peat was considered the optimal method for the amelioration of the coastal saline soil in the study area. The results could be useful for the formulation of strategies toward water conservation and simultaneous desalinization of saline soils in estuarine coastal zones similar to the Yellow River Delta.

##  Supplemental Information

10.7717/peerj.11766/supp-1 Supplemental Information 1 Raw data

## Additional Information and Declarations

### Data Availability

The following information was supplied regarding data availability: The raw measurements are provided in the Supplementary File.
